# Human biting mosquitoes and implications for West Nile virus transmission

**DOI:** 10.1186/s13071-022-05603-1

**Published:** 2023-01-02

**Authors:** Johnny A. Uelmen, Bennett Lamcyzk, Patrick Irwin, Dan Bartlett, Chris Stone, Andrew Mackay, Arielle Arsenault-Benoit, Sadie J. Ryan, John-Paul Mutebi, Gabriel L. Hamer, Megan Fritz, Rebecca L. Smith

**Affiliations:** 1 Department of Pathobiology, College of Veterinary Medicine, University of Illinois at Urbana-Champaign, 3505 Veterinary Medicine Basic Sciences Building, 2001 S. Lincoln Ave, Urbana, IL 61802 USA; 2 Northwest Mosquito Abatement District, 147 W. Hintz Rd, Wheeling, IL 60090 USA; 3 Illinois Natural History Survey, Prairie Research Institute, University of Illinois at Urbana-Champaign, Forbes Natural History Building, 1816 S. Oak Street, M/C 652, Champaign, IL 61820 USA; 4 Department of Entomology, College of Computer, Mathematical, and Natural Sciences, University of Maryland, 4112 Plant Sciences Building, College Park, MD 20742 USA; 5 Department of Geography, College of Liberal Arts and Sciences, University of Florida, 3141 Turlington Hall, 330 Newell Dr, Gainesville, FL 32611 USA; 6 Division of Vector-Borne Diseases, Arboviral Disease Branch, US Centers for Disease Control and Prevention, 3156 Rampart Rd., Fort Collins, CO 80521 USA; 7 Department of Entomology. College of Agriculture & Life Sciences, Texas A&M University, TAMU 2475, College Station, TX 77843 USA

**Keywords:** Human landing catch, *Culex salinarius*, West Nile virus, Zoonosis, Vector-borne disease, Spillover

## Abstract

**Background:**

West Nile virus (WNV), primarily vectored by mosquitoes of the genus *Culex*, is the most important mosquito-borne pathogen in North America, having infected thousands of humans and countless wildlife since its arrival in the USA in 1999. In locations with dedicated mosquito control programs, surveillance methods often rely on frequent testing of mosquitoes collected in a network of gravid traps (GTs) and CO_2_-baited light traps (LTs). Traps specifically targeting oviposition-seeking (e.g. GTs) and host-seeking (e.g. LTs) mosquitoes are vulnerable to trap bias, and captured specimens are often damaged, making morphological identification difficult.

**Methods:**

This study leverages an alternative mosquito collection method, the human landing catch (HLC), as a means to compare sampling of potential WNV vectors to traditional trapping methods. Human collectors exposed one limb for 15 min at crepuscular periods (5:00–8:30 am and 6:00–9:30 pm daily, the time when *Culex* species are most actively host-seeking) at each of 55 study sites in suburban Chicago, Illinois, for two summers (2018 and 2019).

**Results:**

A total of 223 human-seeking mosquitoes were caught by HLC, of which 46 (20.6%) were mosquitoes of genus *Culex*. Of these 46 collected *Culex* specimens, 34 (73.9%) were *Cx. salinarius*, a potential WNV vector species not thought to be highly abundant in upper Midwest USA. Per trapping effort, GTs and LTs collected > 7.5-fold the number of individual *Culex* specimens than HLC efforts.

**Conclusions:**

The less commonly used HLC method provides important insight into the complement of human-biting mosquitoes in a region with consistent WNV epidemics. This study underscores the value of the HLC collection method as a complementary tool for surveillance to aid in WNV vector species characterization. However, given the added risk to the collector, novel mitigation methods or alternative approaches must be explored to incorporate HLC collections safely and strategically into control programs.

**Graphical Abstract:**

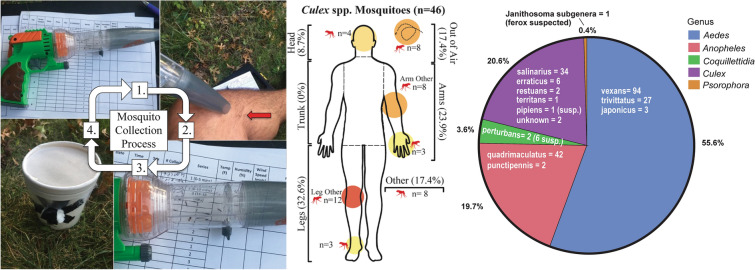

**Supplementary Information:**

The online version contains supplementary material available at 10.1186/s13071-022-05603-1.

## Background

West Nile virus (WNV) is a zoonotic mosquito-borne *Flavivirus* naturally maintained in a mosquito-bird-mosquito enzootic cycle [1, 2]. The risk of spillover to humans increases with greater exposure to several primary vector species of WNV within the *Culex* genus. Since the arrival of WNV to the USA in 1999, there have been 52,532 reported human infections, accounting for 2456 (4.7%) deaths from the infection [3, 4]. More than two decades after its arrival, WNV remains the most important mosquito-borne pathogen in North America [5]. Now endemic, WNV persists throughout the USA, even in locations with operational area-wide vector control [6]. Effective mosquito control is augmented by a robust surveillance program based on traps gathering data on vector abundance and infection with WNV [7].

Gravid traps (GTs) and CO_2_-baited light traps (LTs) are the most common tools for WNV surveillance used in the USA [8–10] and are extremely effective at collecting a large number of mosquito specimens per trapping effort; GTs tend to collect large numbers of mosquitoes belonging to genus *Culex* while LTs trap a greater diversity of mosquito species. Mosquitoes trapped in GTs and LTs often consist of large collections spanning multiple mosquito genera, as well as a diversity of other non-Culicid insect species [11]. The large size and high diversity of these sample collections can make sorting and identifying WNV vector species labor intensive. Adult mosquitoes collected from GTs and LTs are usually sorted by sex (with male specimens often discarded) and identified by key morphological features upon examination under a dissecting microscope. Mosquitoes captured in these types of traps pass through a bladed fan and often get damaged, or they become desiccated (in dry climates) or moldy (in humid climates) if not collected within 1 or 2 days [12]. The methods of identification, in combination with high volumes of collected mosquitoes and difficulties in discerning subtle features across several key *Culex* species, are often prone to misidentification from human error [13, 14]. In addition to members of the *Culex pipiens* complex, *Culex restuans, Culex salinarius* and even *Culiseta inornata* specimens can be misidentified as *Cx. pipiens* or simply lumped into the term “*Culex* species” [15].

Deployment of a network of GTs and LTs, frequent mosquito collections and rapid pathogen testing are considered the “status quo” for understanding mosquito abundance and WNV infection. When a combination of high abundance and high infection rates indicates increased human risk, mosquito control agencies will often then employ control efforts, such as adulticide spraying or larvicide deposition [16, 17]. These control methods are noticeably effective in the immediate days following action. However, evidence from northern Illinois suggest that impacts on mosquito abundance from spray control methods may only be temporary, and only within a fairly small radius of the area of treatment, and that populations recover rapidly, usually in less than 1 week [18, 19]. While the overall rapid reduction of all mosquitoes may be a goal for mosquito abatement agencies [20] and citizens [21], the key public health emphasis in relation to WNV surveillance and control should be to monitor and control species of mosquitoes involved in the enzootic transmission and spillover to humans.

Given ongoing and persistent difficulties controlling mosquito populations and eliminating WNV from the environment, as well as the current limitations to predictive models of human WNV cases, we investigated whether adding information from the human landing catch (HLC) method to data obtained using traditional mosquito collection methods already in place to protect public health from arboviral threats can improve targeted WNV surveillance and control efforts. A long-term WNV transmission research effort in the northwest suburbs of Chicago, state of Illinois (USA), in close collaboration with the Northwest Mosquito Abatement District (NWMAD), has worked to pinpoint missing links between human WNV illness and mosquito infection at multiple spatial scales, including highly localized study sites [6, 22, 23]. The goal of the present study was to use the HLC collection method (considered to be the gold standard for assessing mosquito-human host interactions [24]) to evaluate the effectiveness in attracting *Culex* species mosquitoes targeting human blood meals at locations where there has previously been a high incidence of human illness [25, 26]. We then compared the HLC data with traditional mosquito surveillance data collections using GTs and LTs and determined key differences among the collected mosquito genus and species and in the relative proportion of abundance. We hypothesized that the HLC collection method would provide a higher relative proportion of human-seeking mosquitoes, particularly among *Culex* species, thereby improving our understanding of potential vectors involved in WNV spillover to humans in the NWMAD. Insights into the interactions between humans and WNV vector species may lead to improved targeted surveillance for WNV vector species that seek human blood meals.

## Methods

### Ethics statement

This project was approved by the Institutional Review Board (approval number 18908) of the University of Illinois at Urbana-Champaign, the Illinois Department of Public Health (IDPH) and the University of Illinois Biosafety Committee (approval number IBC-4307). All HLC participants were researchers and were informed of and educated on the potential risks of the study prior to field collecting.

### Study sites

Mosquitoes collected via HLCs and human observations were conducted within the NWMAD, a mosquito control agency area, approximately 240 square miles in size, comprising the northwest suburbs of Chicago. Previous research had established 55 1-km wide hexagonal study locations within the NWMAD, thereby providing representative focal study regions selected through stratification of high to low categorization values of previous human WNV risk (high to low) and prior modeling accuracy (high to low residual of predictions) [6, 22]. Additionally, data on key socioeconomic parameters (average racial composition, housing age and income) specific to each hexagonal study area were available to include in the analyses. Within each hexagonal region, a natural area (e.g. public park) was selected as a collection site because such areas were easily accessible and allowed for collections during crepuscular periods (5–8:30 am and/or 6–9:30 pm) when *Culex* mosquitoes are most actively seeking blood meals [27, 28]. The sites also met the study criteria for locations where mosquito-human spillover likely occurs in the Midwest USA for two reasons: (i) all locations were composed of heterogeneous landscapes consisting of a mix of domestic and peri-domestic built space, a body of water (e.g. pond, river) and natural green spaces rich with numerous avian species [29, 30]; and (ii) humans were abundant and engaging in a variety of activities (e.g. resting to physical activity).

### Human landing catches

**Fig. 1:**
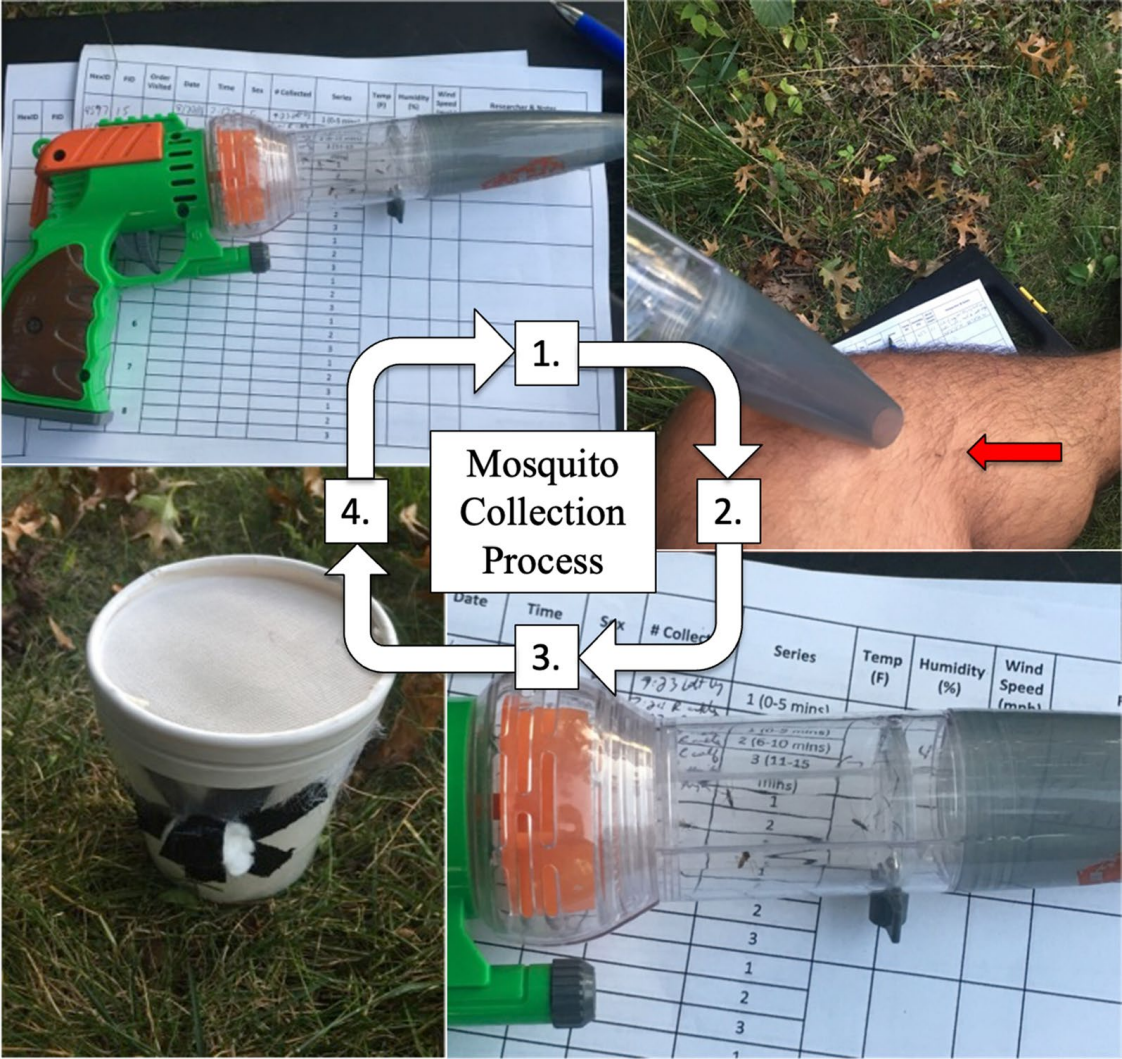
Setup and equipment used for human landing catches. A mechanical aspirator (*1*) was used to collect mosquitoes that landed on an exposed human collector’s limb (*2*). Mosquitoes were aspirated prior to taking a blood meal and contained within the aspirator’s collection chamber (*3*) and then transferred to a cup designed to fit the aspirator’s nozzle (*4*). Cups were then placed in a freezer at − 80 °C and later identified

Human landing catches were conducted between epidemiological weeks (epi-week) 28 and 38 (early July–mid September) in the summers of 2018 and 2019. During these periods, each of the 55 1-km wide hexagonal study regions within the NWMAD was visited weekly for 15 min each visit. Additionally, the visiting order was rotated each week so that all sites were equally sampled at earlier and later times of the crepuscular period; a consistent number of sites (*n* = 27–28) were visited each day. Human collectors (*n* = 4) were consenting student/employees of the University of Illinois, and all had prior experience with arthropod vector biology and field collections. Each of the four human collectors exposed any one to four limb(s) and collected landing mosquitoes via a mechanical aspirator (Fig. 1). Collectors were instructed not to apply any materials to their clothing or body that might be attractive or repellent to host-seeking mosquitoes (e.g. mosquito repellent, strong smelling deodorant, hair spray/gel, cologne, etc.). Mosquitoes were collected within 3–4 s after landing on the collector’s exposed skin. Over a 15-min collection period, mechanically aspirated mosquitoes were transferred to a pre-made paper cage and maintained live until transferred to a freezer and stored at − 80 °C.

Within 3 days, all mosquitoes were removed from the freezer and morphologically identified by medical entomologists at the University of Illinois, referencing identification keys by Craker and Collins [31] and Darsie and Ward [32]. Mosquitoes from the *Culex* genus were sent to the University of Maryland for morphological and molecular confirmatory species identification. These mosquitoes were shipped overnight on dry ice to the University of Maryland, College Park. Upon receipt, they were kept at − 80 °C. Mosquitoes were dissected and abdomens retained as a voucher. Genomic DNA was extracted from heads and thoraces with a Qiagen DNeasy Blood and Tissue Kit (cat. no. 69506; Qiagen Inc., Valencia CA, USA) according to the standard protocol. Extracted DNA was amplified in a multiplex PCR assay targeting the 28S ribosomal subunit [33] to distinctly identify *Cx. pipiens* (698 bp)*, Cx. restuans* (506 bp) and *Cx. salinarius* (175 bp), using a reaction similar to that described in [34], except that the total reaction volume was 20 μl, and we used the MgCl_2_ concentration that is standard in the Promega GoTaq buffer (Promega, Madison, WI, USA). A negative control, in which purified water replaced genomic DNA, was run with mosquito samples during each assay. The PCR assay was run using a Bio-Rad T100 Thermal Cycler (Bio-Rad Laboratories, Hercules, CA, USA) at the following conditions: 96 °C for 4 min, 35 cycles of 96 °C for 15 s, 55 °C for 30 s, 72 °C for 90 s, then 72 °C for 4 min. We visualized amplicons following gel electrophoresis in a 2% agarose gel at 120 V for 60 min, and specimens were identified based on fragment length, using a 1-kb ladder (Gene Ruler 1 kb Plus; Thermo Fisher Scientific, Waltham, MA, USA) for comparison.

### Human observations

While HLC collections were conducted by a human collector, an accompanying researcher simultaneously recorded the number of unique human visitors within eyesight at the same location. Uelmen [35] provides additional data on the unique activities, duration of activities and apparent age and gender of humans in terms of assessing and quantifying WNV risk and human behavior.

Using data from HLCs, as a proxy for mosquito biting rates, and human observations, as a proxy for human host availability, we derived two indices: the nuisance factor and the human WNV added risk factor. The majority of collected mosquitoes were non-*Culex* species and less likely to vector WNV and other regional mosquito-borne pathogens to humans. These non-*Culex* species were thus considered to be nuisances to the public, and the nuisance factor index defined as:

$$Nuisance \, Factor=\frac{\frac{Human \, Observations}{Hour}*\frac{Nuisance \, Mosquitoes \, Collected}{Hour}}{100}$$

Conversely, the human WNV added risk factor was defined for collected mosquitoes of the *Culex* genus, by the following equation:

$$Human \, WNV \, Added \, Risk= \frac{\frac{Human \, Observations}{Hour}* \frac{Culex\mathrm{ species \, Collected}}{\mathrm{Hour}}}{100}$$

The denominator is used for ease of interpretation and visualization. All observed humans were assumed to be equally available to mosquitoes, regardless of activity and/or behavior.

### Historical mosquito collections

**Fig. 2:**
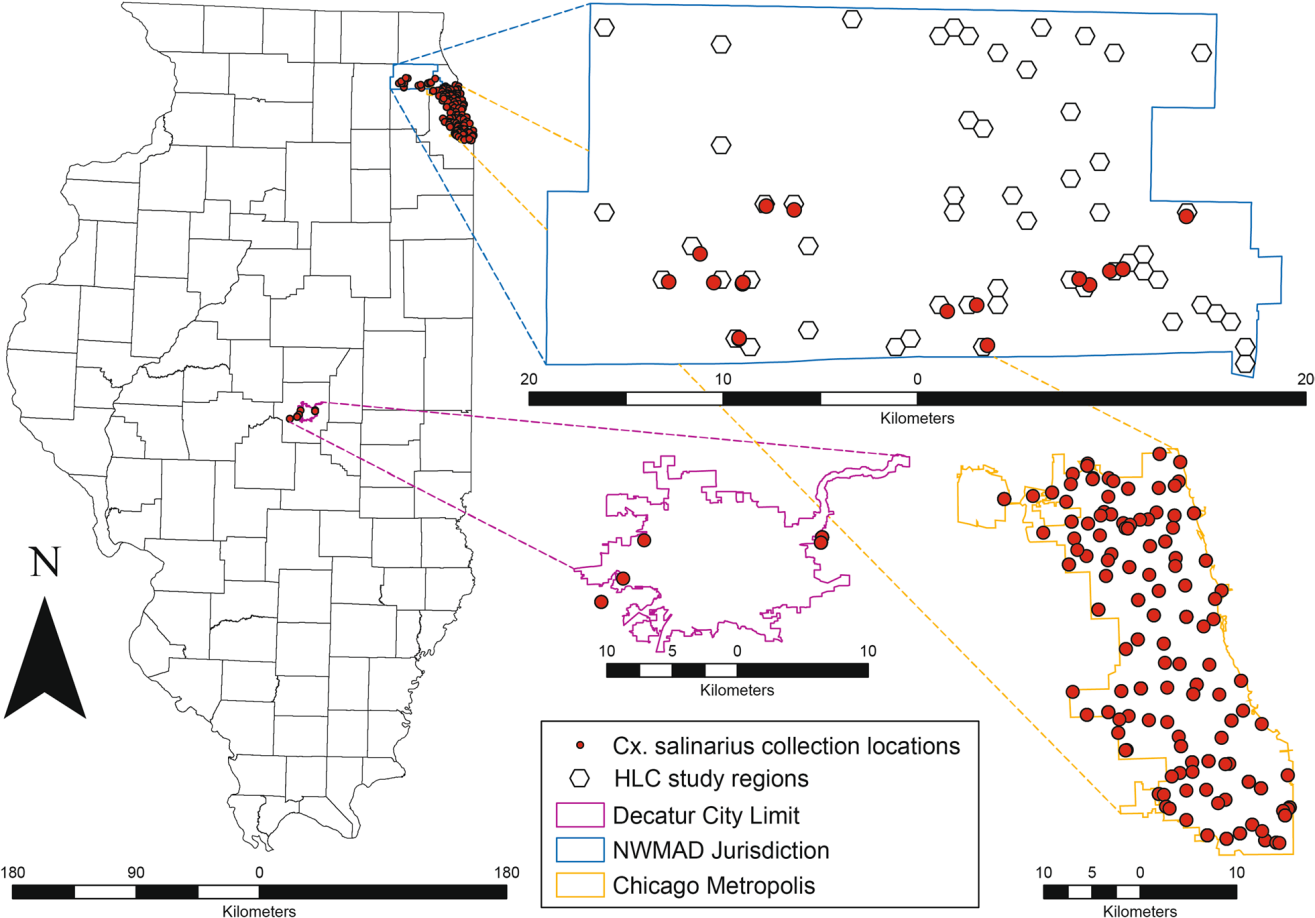
Locations of *Culex salinarius* collected within Illinois. Human landing catch (HLC) efforts (conducted within each of the 55 1-km wide hexagonal units) totaled 34 collections from 4.32 trap nights. The City of Chicago, including the Northwest Mosquito Abatement District (NWMAD) (all non-HLC trapping methods) and southern Cook County collections, resulted in a total of 1466 specimens trapped over a combined 77,062 trap nights. Macon Mosquito Abatement District totaled 199 collections from 9299 trap nights

Historical data from mosquito collections of the US Centers for Disease Control and Prevention (CDC) GTs and from New Jersey LTs (John Hock Company, Gainesville, FL, USA) from four sources were examined, three from Chicago and surrounding suburbs, and another from Decatur, Illinois (Fig. 2). Trapping effort was standardized as a combined trap night, a sum of the total number of night GTs and LTs that were set to collect mosquitoes. The relative abundance of *Culex* and non-*Culex* species mosquitoes were compared with HLC collections conducted in the NWMAD.

Public health agencies serving the city of Chicago and the surrounding suburbs in Cook and DuPage Counties are among the best equipped agencies (for example, in terms of large annual budgets, number of personnel and available equipment and tools) to combat mosquitoes and mosquito-borne diseases in the country. There are four dedicated abatement districts that serve the surrounding suburbs, as well as a vector control branch of the Chicago Public Health Department that serves the greater metropolitan area. The NWMAD and the Chicago Vector Control Services provided surveillance records on the presence of *Cx. salinarius* in the greater Chicago area. In addition to these agency-provided data, collections conducted in the suburbs of southern Cook County (Hamer et al., unpublished) were included. The mosquito abatement district of Macon County (MMAD), located in central Illinois, provided surveillance records for the greater Decatur region, serving as an excellent comparator to the abundance and spectrum of mosquitoes genus and species between the two regions.

### Statistical analyses

Descriptive analyses were conducted using univariate tests for socioeconomic and demographic drivers of *Culex* species collected via HLC methods (Additional file 1: Tables S1 & S2). The mean of mosquito phenology and biting time of day were assessed by Tukey’s honest significance test (HSD). Mosquito abundance, genus and species and WNV illness risk were compared by trapping effort (ratio of trap nights to total mosquitoes by genus) and type of trap (HLC, GT, LT, other/unknown) and assessed as multinomial distributions in a generalized linear regression. All statistical analyses were conducted in JMP (version 16.0.0; SAS Institute Inc., Cary, NC, USA) and R (version 4.1.2.).

## Results

### HLC collections

**Fig. 3:**
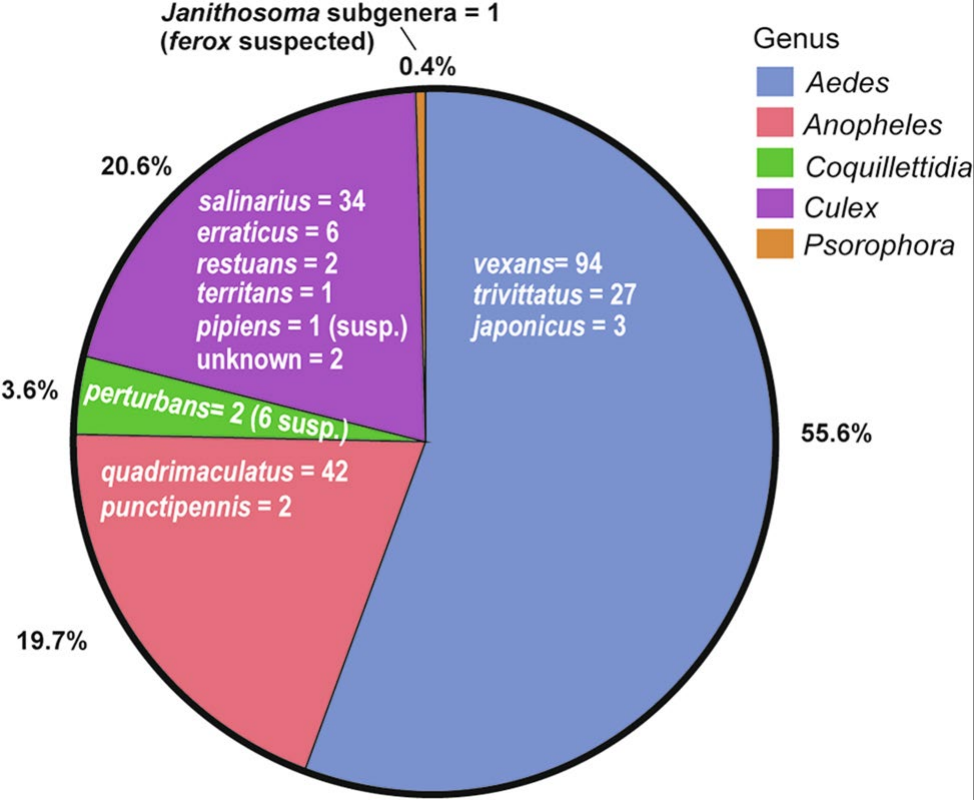
Results from HLC efforts over two summers (2018 and 2019) within the NWMAD, resulting in a total catch of 223 mosquitoes representing 12 species across 5 genera. Additional figures displaying historical mosquito collections are located in Additional file 1: Figures S2, S3

A total of 223 mosquitoes were collected by HLC, including 46 (20.6%) *Culex* specimens (Additional file 1: Figure S1). This amounted to a catch rate of 51.6 mosquitoes of all species and 10.7 mosquitoes of genus *Culex*, per trap night. Mosquitoes belonging to genus *Aedes* were the most abundant (55.6%), followed by those belonging to the genus *Culex* (20.6%), *Anopheles* (19.7%)*, Coquillettidia* (3.6%) and *Psorophora* (0.4%) (Fig. 3). The most abundant species collected were *Aedes vexans* (42.2%)*, Anopheles quadrimaculatus* (18.8%)*, Cx. salinarius* (15.2%) and* Aedes trivittatus* (12.1%) (Additional file 1: Figure S4).

**Fig. 4:**
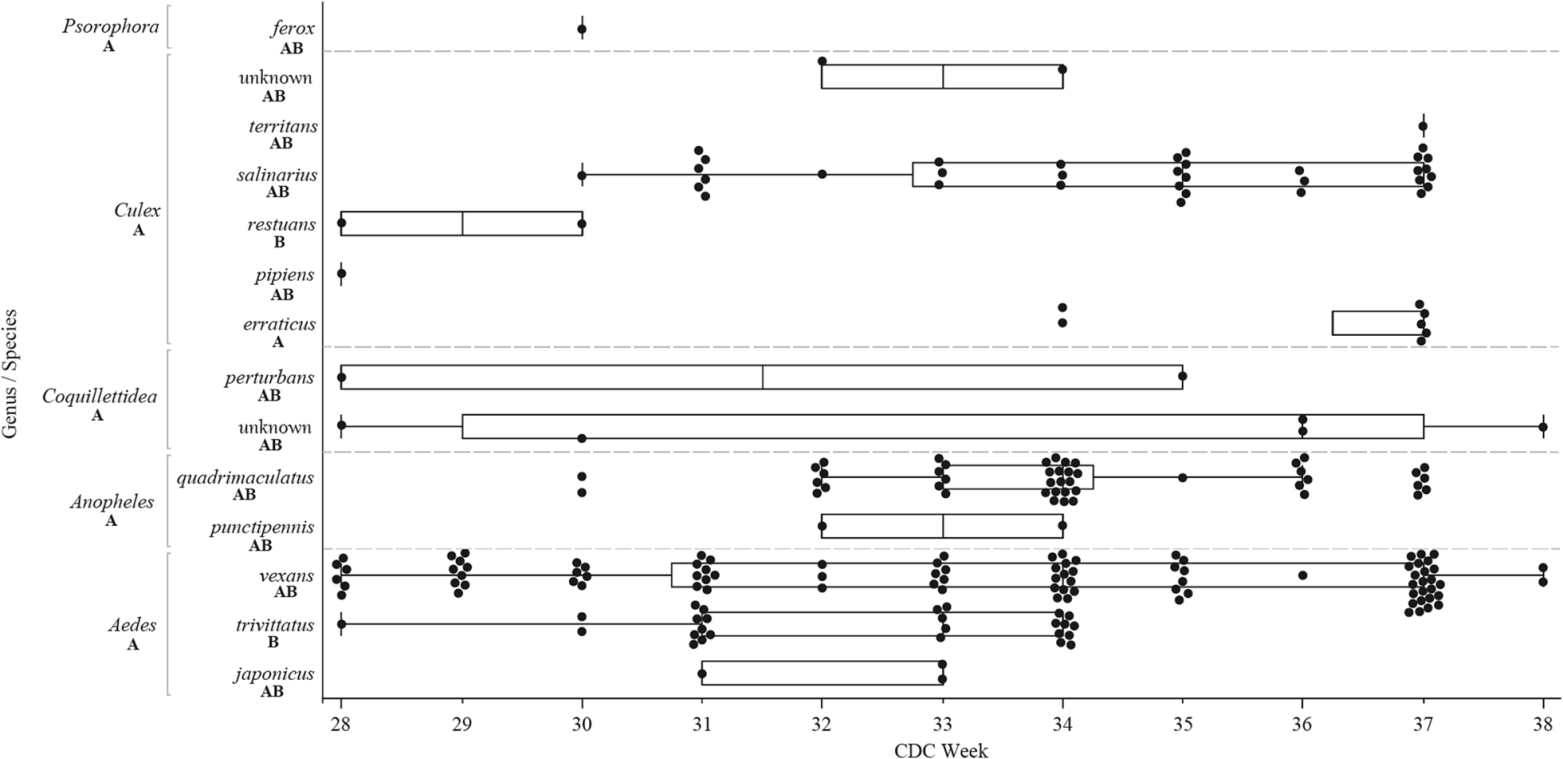
Box plot distributions (median, quartiles and outliers) of mosquito catch by genus and species, caught via HLC by CDC epidemiologic week (CDC Week). HLC collections occurred within the 55 hexagonal study regions of the NWMAD over the summers of 2018 and 2019. Each dot represents an individually collected mosquito. Unique uppercase letters below genus/species names indicate significantly different groupings, as designated by Tukey's honest significance test (HSD)

Mosquito landing rates significantly varied by epi-week according to genus (*P* = 0.036, Tukey's HSD). By species across collection season, *Cx. erraticus* landing rates tended to be significantly later (mean epi-week: 36; *P < *0.05, Tukey's HSD), and *Cx. restuans* and *Ae. trivittatus* landing rates tended significantly earlier (mean epi-weeks: 29 and 33, respectively; *P < *0.05), compared to all other species (Fig. 4).

**Fig. 5:**
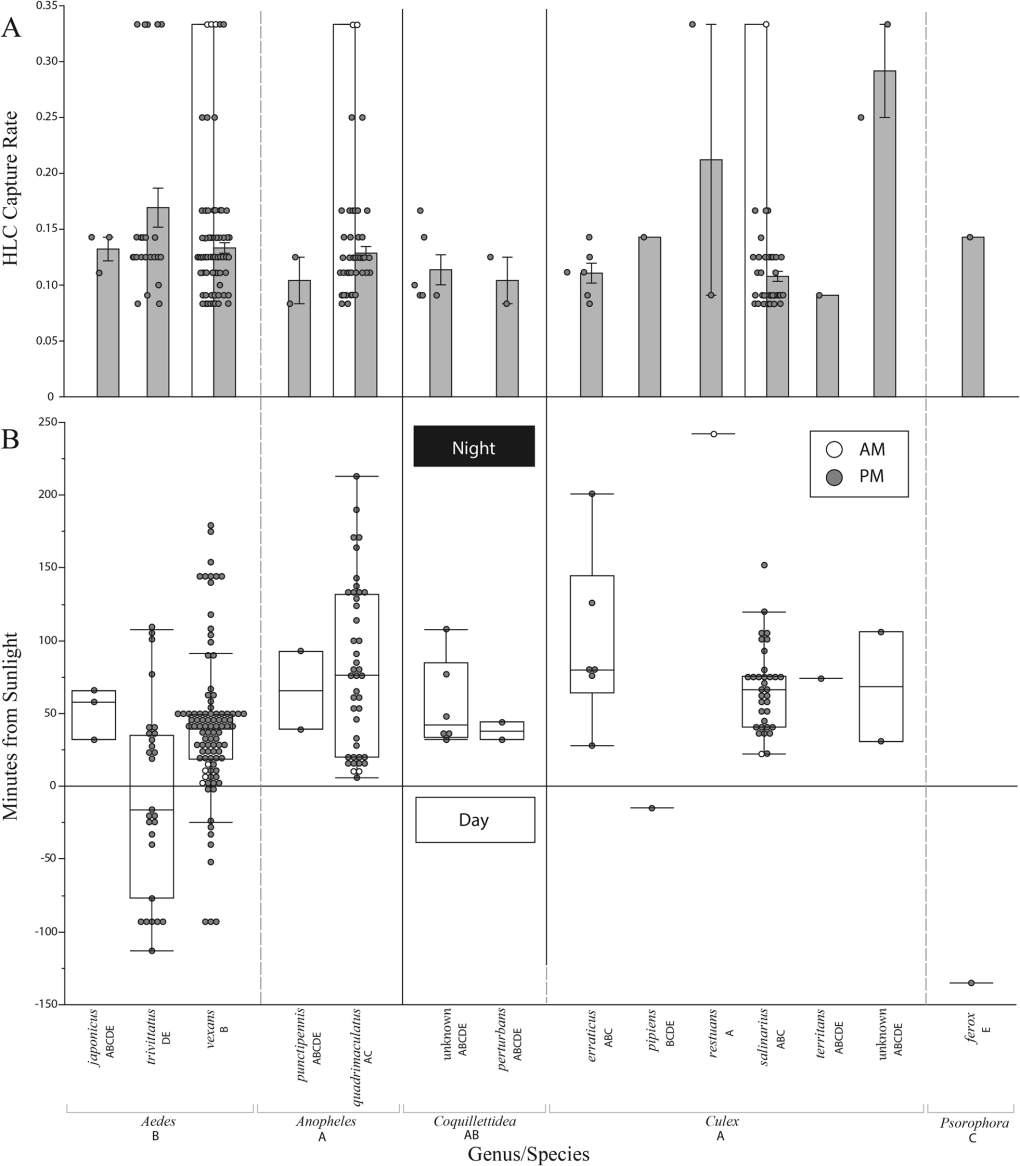
Human landing catch collections by mosquito genus as a function of HLC capture rate (**a**) and minutes from sunlight (**b**) by photoperiod (am vs pm). HLC capture rates were calculated as ($\frac{\sum \, unique \, species \, collected}{\sum \, all \, species \, collected}$) across all collections. Mosquitoes below the horizontal line were collected in the daytime (between sunrise and sunset) while mosquitoes above the line were collected at nighttime (between sunset and sunrise). A total of 8 mosquitoes were collected in the am hours, while the remainder were collected in the pm hours. Unique uppercase letters below genus/species names indicate significantly different groupings, as designated by Tukey's HSD

Mosquito landing rates varied by time of day by genus and species (Fig. 5). Collectively, mosquitoes of genera *Aedes* and *Psorophora* landed significantly earlier in the day (peaking at 30 and 120 min before sunset, respectively) while those of genera *Culex and Anopheles* landed significantly later in the evening hours (peaking at 70 min after sunset for each; *P < *0.05, Tukey's HSD). By individual species, only *Cx. restuans, Ae. vexans* and *Psorophora ferox* differed significantly in landing rates from the other species, peaking at 250 min after, 50 min after and 120 min before sunset, respectively (*P < *0.05, Tukey's HSD) (Table 1). Landing rates per HLC night did not statistically differ by researcher (*P* = 0.0610), genus (*P* = 0.8769) or species (*P* = 0.8652, GLMTable 2).

**Table 1:** Mosquito collection data according to each reporting location per trap night, trapping method and total collections by year

Mosquito collection data by each reporting location per trap night											
Location	Trapping years	Trapping nights all methods	Total mosquitoes collected								
			All species			*Culex* species			*Culex salinarius*		
			Sum	Per year	Per trap night	Sum	Per year	Per trap night	Sum	Per year	Per trap night
Ultra-fine-scale NWMAD: 55 hexagonal study regions (HLC)	2018–2019	4.32*	223	111.5	51.62	46	23	10.65	34	17	7.87
NWMAD	2005–2016	67,763	1,580,526	131,710.5	23.32	1,169,168	97,430.67	17.25	548	45.67	0.01
Southern Cook County, IL	2005–2012	N/A	5414	676.75	N/A	2222	277.75	N/A	22	2.75	N/A
City of Chicago	2005–2019	N/A							896	59.73	
MMAD	2002–2018	9299	344,517	20,265.706	37.05	305,021	17,942.41	32.8	199	11.71	0.02
Total	54	77,066.32	1,930,680	152,764.46	111.99	1,476,457	115,673.83	60.7	1699	136.86	7.9

Mosquito collection data provided by each reporting location by trapping method																
Location	Trapping years	Non-*Culex* species					*Culex* species									
							All species					*Culex salinarius*				
		Sum	LT	GT	HLC	Other	Sum	LT	GT	HLC	Other	Sum	LT	GT	HLC	Other
Ultra-fine-scale NWMAD: 55 hexagonal study regions (HLC)	2018–2019	177	0	0	177	0	46	0	0	46	0	34	0	0	34	0
NWMAD	2005–2016	411,358	N/A				1,169,168	59,127	1,110,041	N/A	N/A	548	548	N/A	N/A	N/A
Southern Cook County, IL	2005–2012	3192	N/A				2222	276	1433	0	513	22	18	4	N/A	
City of Chicago	2005–2019	N/A										896	390	443	0	63
MMAD	2002–2018	39,496	N/A				305,021	16,374	288,647	N/A		199	196	3	N/A	
Total	54	454,223	0	0	177	0	1,476,457	75,777	1,400,121	46	513	1699	1152	450	34	63
% *Culex salinarius*	–	–	–	–	–	–	0.12%	1.52%	0.03%	73.91%	12.28%					

Total mosquito collection data by year									
Year	Ultra-fine-scale NWMAD 55 hexagons	NWMAD		Southern Cook County, Illinois			City of Chicago	MMAD	
	HLC	LT	GT	LT	GT	Other*	HLC	LT	GT
2002								1001	57
2003								667	1568
2004								2300	3138
2005		4733	111,198	36	303	49	100	3925	4510
2006		6773	148,130	31	269	244	308	2144	9530
2007		130	69,714	46	206	44	80	1867	12,761
2008		6825	64,109	48	192	0	21	1419	7627
2009		9746	49,387	15	106	88	42	423	13,373
2010		5543	54,267	53	151	44	135	57	11,280
2011		5059	47,630	464	1232	320	54	35	9794
2012		7834	109,248	26	296	8	26	231	10,204
2013		5214	179,160				5	115	19,938
2014		3489	117,913				14	60	33,385
2015		2420	116,139				35	34	32,295
2016		1361	43,146				6	179	44,666
2017							4	17	33,809
2018	8						57	1900	40,712
2019	38						9		

Detailed information on the collection by genus is given in Additional file 1

*HLC* Human landing catch,* GT* gravid trap,* LT* light trap,* MMAD* Macon Mosquito Abatement District,* N/A* not available,* NWMAD* Northwest Mosquito Abatement District

**Table 2:** Human landing catch mosquito collections for each researcher by species and overall

HLC collections for each researcher by species			
Researcher	HLC nights	Number of mosquitoes collected	Mosquitoes per HLC night
1	12	34	2.83
2	16	34	2.125
3	21	174	8.29
4	4	3	0.75

HLC collections overall					
Researcher	Mosquito genus	Mosquito species	HLC nights	Number of mosquitoes collected	Mosquitoes per HLC Night
1	*Aedes*	*Japonicus*	2	2	1
		*Trivittatus*	1	1	1
		*Trivittatus*	2	9	4.5
		*Vexans*	3	14.5	4.83
	*Anopheles*	*Punctipennis*	1	0.5	0.5
		*Quadrimaculatus*	2	5.5	2.75
	*Culex*	*Salinarius*	1	1.5	1.5
2	*Aedes*	*Japonicus*	1	1	1
		*Trivittatus*	2	2	1
		*Vexans*	3	10	3.33
	*Anopheles*	*Punctipennis*	1	0.5	0.5
		*Quadrimaculatus*	2	9.5	4.75
	*Coquillettidia*	*Perturbans*	1	0.5	0.5
	*Culex*	*Erraticus*	1	1	1
		*Pipiens*	1	0.5	0.5
		*Restuans*	1	0.5	0.5
		*Salinarius*	2	7.5	3.75
	*Psorophora*	*Janthisoma*	1	1	1
3	*Aedes*	*Japonicus*	1	1	1
		*Trivittatus*	2	7	3.5
		*Trivittatus*	3	17	5.67
		*Vexans*	4	79	19.75
	*Anopheles*	*Punctipennis*	1	1	1
		*Quadrimaculatus*	3	28	9.33
	*Coquillettidia*	*Perturbans*	1	7	7
	*Culex*	*Erraticus*	1	5	5
		*Restuans*	1	1	1
		*Salinarius*	2	25	12.5
		*Territans*	1	1	1
		*Unknown*	1	2	2
4	*Aedes*	*Vexans*	1	1.5	1.5
	*Coquillettidia*	*Perturbans*	1	0.5	0.5
	*Culex*	*Pipiens*	1	0.5	0.5
		*Restuans*	1	0.5	0.5

Mosquitoes collected per HLC night did not statistically differ by researcher or species

### Human activity and risk

**Fig. 6:**
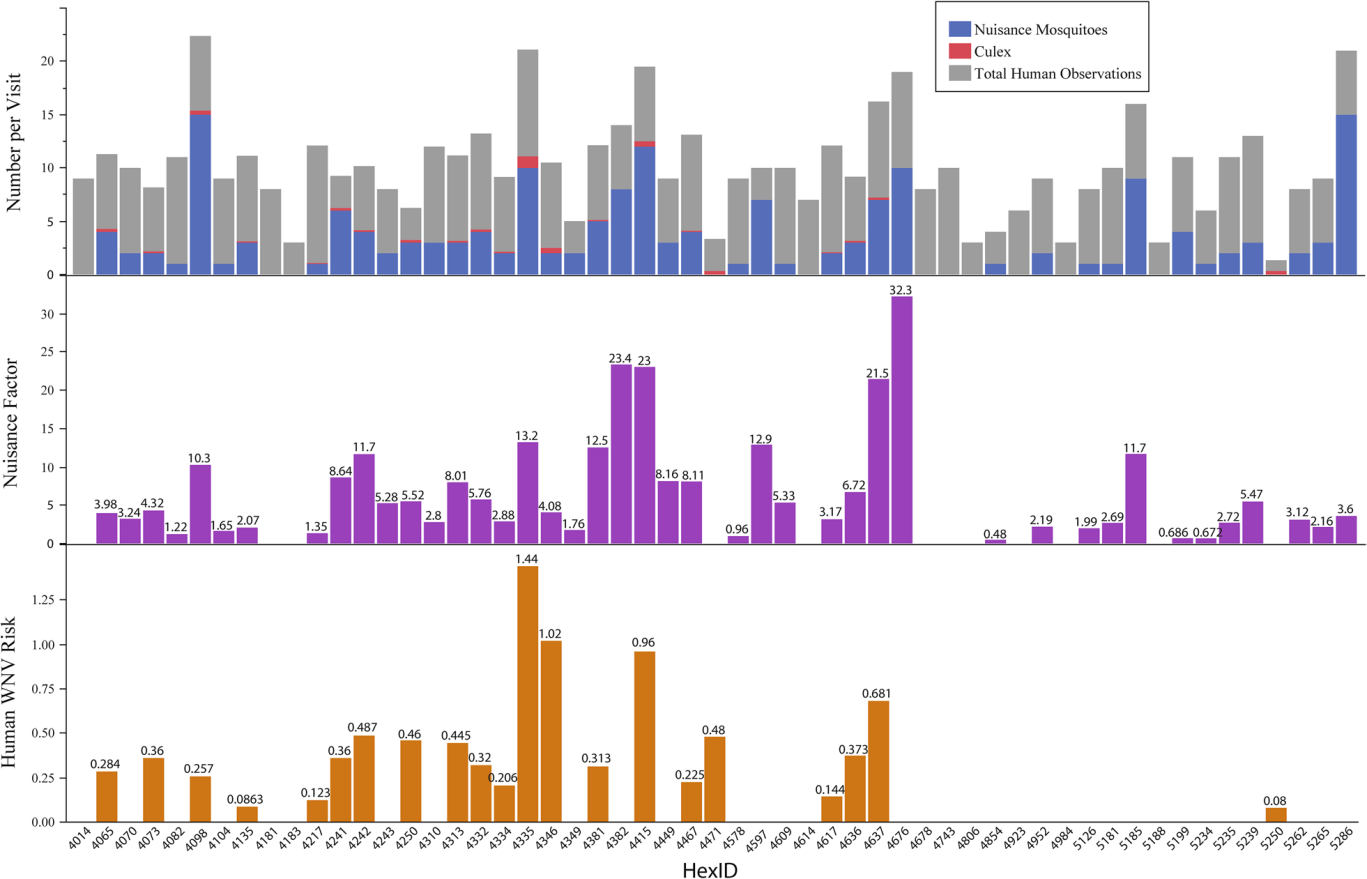
Frequency of human activity observations and total number of mosquitoes collected by type (nuisance and *Culex* species) (top) combined to create two indices: the nuisance factor (middle) and the WNV added risk (bottom). Detailed information regarding these indices is provided in [6]. WNV, West Nile virus

Human observations were recorded at every collection location, but nuisance mosquitoes were not present in 12 of the 55 locations and *Cx.* mosquitoes were not present in 33 of the 55 study locations (Fig. 6). A total of 2821 individual humans were counted over 2040 recording minutes (mean: 1.4 people per minute) during HLC collections. By recording location, human observations per 15-min visit ranged from a maximum of 312 to a minimum of 1. The nuisance factor ranged from a maximum of 32.3 to a minimum of 0, and the human WNV added risk factor ranged from a maximum of 1.44 to a minimum of 0.

### Historical mosquito abundance

A compilation of historical abundance records resulted in a total of 1,930,680 collected mosquitoes (all species) across the four historical data sources (Table 1;Additional file 1: Figure S3). Of these, 454,223 (23.5%) were non-*Culex* species, and 1,476,457 (76.5%) were *Culex* species. MMAD provided the longest period of collections (17 years), but the NWMAD had the greatest number of mosquitoes collected (*n* = 1,169,168; 60.6%). Per trapping effort, standardized as number of mosquitoes collected per trap night, MMAD produced the most mosquitoes (37.1 per trap night) over the 17-year collection period.

Overall, *Culex* species mosquitoes were collected in greater numbers in GTs (*n* = 1,400,121; 94.8%) than in LTs (*n* = 75,777, 5.1%) (Table 1B; Additional File 1: Figure S4). Per combined LT and GT effort, more *Culex* species were caught in MMAD (32.8 per trap night) than in NWMAD (17.3 per trap night). The ratios of *Culex* species mosquitoes collected from LT to those collected from GT in NWMAD and MMAD were similar (0.053 and 0.057, respectively); from Southern Cook County traps, the same ratio was threefold higher (0.193).

## Discussion

### HLC comparisons to historical records

Each year high volumes of mosquitoes are caught in GTs and LTs in the NWMAD and MMAD, including tens of thousands of potential WNV vectors. Since 2002, many human cases of WNV illness have occurred in the city of Chicago annually, despite rigorous mosquito control campaigns and the efforts of dedicated abatement and local health districts working year-round [6]. Evaluation of mosquito species collections obtained using different trapping methods is not precise in terms of determining abundances in a region due to trap-specific biases, lack of a systematic collection regimen and non-uniform distribution of equally placed trap types in the study region.

Based on a rich body of historical trapping data that cover the past decade, this study provides a reasonable baseline by which *Culex* species, and *Cx. salinarius* in particular, are expected to be collected. Mosquitoes of the genera *Aedes, Culex, Anopheles, Coquillettidia* and *Psorophora* accounted for 55.6%, 20.6%, 19.7%, 3.6% and 0.4% of the landing catch, respectively. The proportions of *Aedes, Anopheles, Coquillettidia* and *Psorophora* in collections from NWMAD and MMAD were 21.1% and 10.2%, 0.6% and 1.0%, 0.5% and 0.07% and 0.01% and 0.2%, respectively. In contrast, proportions of *Culex* species collected from NWMAD and MMAD were 70.2% and 83.8% in GTs, and 3.7% and 4.8% in LTs, respectively. Overall, HLCs collected higher proportions of non-*Culex* mosquitoes than GTs or LTs. In a separate study, 785 mosquitoes of various species of genus *Culex* from the North Shore District and NWMAD of Northern Cook County, Illinois were collected from GTs and LTs between 2017–2021 and submitted for confirmatory genetic species identification (Fritz et al., unpublished). Only two (0.25%) of these specimens were identified as *Cx. salinariu*s, compared to 73.9% of specimens from HLCs. Thus, the expected distribution of species collected will depend on the collection method.

Traditional trapping methods are ideal for the efficient collection and testing of potential WNV mosquito vectors. However, these methods are only effective in identifying potential vectors and controlling virus activity if testing is conducted frequently because *Culex* populations are known to rebound rapidly after spraying [18]. Conversely, traditional trapping methods appear to collect a limited diversity of mosquito species [36, 37]. One possible explanations for this is that other mosquito species may not have a biological preference or attraction to the simulated baiting conditions (e.g. lights, CO_2_, lures, etc.), but a more likely reason is resource strain (e.g. limited human time to sort and identify mass quantities of mosquitoes). HLC collections do not have the same proportions of *Culex* species that GTs and LTs collect. Additionally, the HLC method has two major disadvantages: (i) it is labor intensive and time consuming, requiring a lot of human resources per captured mosquito; and (2) the collector’s risks of acquiring mosquito-borne pathogens, many of which do not have specific treatments or cures, are increased. However, HLCs are better suited than GTs or LTs for estimating the community of female mosquitoes specifically seeking human hosts [24]. The potential benefits in a systematic and targeted design may pinpoint hotspots for the highest concentration of human-seeking mosquitoes. When used in a targeted-style approach, HLCs can narrow the geographic area of interest and provide a more efficient deployment for controlling potential human-seeking WNV mosquito vectors; however, approaches to safeguard the collector’s health must be further developed before widespread adoption of HLCs for monitoring can be considered [24]. Additionally, the HLCs in this study were conducted in small natural areas, embedded within residential areas. Additional studies are needed to ascertain any influence of HLCs on mosquito ecology within these two intersecting habitats [29]. This study analyzed the results of landing rates based on four human collectors. To evaluate any differences in an individual’s attractiveness, we evaluated the rate of mosquito collections per “HLC night,” as a method for standardizing collection values across varying periods of exposure. These values indicate no statistical difference in an individual’s attractiveness, but future studies should address this question with a targeted design evaluating methods for measuring potential human behaviors, characteristics and/or scents that may be interest. Results from this 2-year study have made clear that *Cx. salinarius*, a less commonly reported WNV vector species in the upper Midwestern USA, was not only present in the Chicago area, but was the most commonly collected WNV vector in HLC collections, accounting for 73.9% of all *Culex* species landings. Conversely, the two primary vectors of WNV in the Midwestern USA, *Cx. pipiens* and *Cx. restuans,* only comprised 4.3% and 2.2% of the total proportion of *Culex* species that landed on human collectors.

### Implications for human WNV transmission

*Culex salinarius* Coquillett mosquitoes, commonly known as the unbanded saltmarsh mosquito, are among the most competent vectors of WNV in laboratory transmission studies [38–40]. The virus has also been frequently isolated from wild-caught specimens [41–45]. The etymology of the name *salinarius* pertains to salt but is slightly misleading given that the species appears to tolerate low to moderate levels of salt (3–11 ppt) in freshwater/brackish environments, particularly in ponds and other small bodies of water along coastlines [46–48]. Historically, the range of this species stretches from Maine to Florida, and around the Gulf of Mexico to Texas [49]. Although specimens of this species have been collected as far north as Ontario, Canada, reports pertaining to its overall abundances in the upper Midwestern USA have remained low, although its presence has been reported from various locations in the Midwest USA [50–54]. Despite *Cx salinarius* and *Cx. pipiens* belonging to the same genus, these two species do not contain high quantities of fat bodies and do not enter a reproductive diapause [55, 56]. Instead, overwintering is thought to occur in natural shelters and animal burrows, and females have been known to seek blood meals at the first signs of mild weather [49]. In addition to not being treated by traditional control methods, the natural habitats that enable the winter survival of *Cx. salinarius* emphasize the importance of incorporating broader vector control measures in different landscapes within urban and suburban environments.

Perhaps the most startling difference between *Cx. salinarius* and *Cx. pipiens* is the host blood meal preference. *Culex pipiens* highly prefers avian hosts, and blood meal analyses have revealed that the proportion of mammalian hosts rarely exceeded 15–20% [2, 57]. In contrast, the host blood meal preference of *Cx. salinarius* exceeded 60% mammalian hosts and between 35 and 40% avian hosts [12, 58–60]. When potential implications for WNV spillover in the Midwestern USA are factored in, *Cx. salinarius* provides three potentially critical points of concern: (i) this species is more commonly observed in its northern range, leading to new questions regarding the species’ northern geographic limit [61], and it is either not commonly trapped with GT and LT methods, or it is not being correctly identified (via morphological methods); (ii) efforts to mitigate potential WNV vectors are not designed for targeting *Cx. salinarius*, given its tendency to breed in natural habitats; and (iii) the high mammalian host preference, aggressive biting nature and high vector competence provide the key ingredients for a mosquito to potentially become a highly efficient bridge vector of WNV and other encephalitis viruses [58, 59, 62].

Upon discovering the high ratio of *Cx. salinarius* landing rates*,* in comparison to those of any other *Culex* species in Chicago, additional data inquiries were made from prior research conducted in the region. Colleagues from the city of Chicago’s vector control program and a collaborative team working in various sites of Southern Cook County shared their abundance data for comparison (Table 1; Additional file 1: Table S1, Table S2). In summary, out of a total of 1,476,411 collected *Culex* specimens, only 1665 (0.11%) were identified as *Cx. salinarius*. HLC efforts resulted in 34 *Cx. salinarius* collections out of a total of 46 *Culex* specimens (73.9%). HLC efforts totaled 7.87 collections per trap night versus 0.022 collections per trap night for all other collection efforts. Taken together, this result equates to HLC collections producing over 364-fold the number of *Cx. salinarius* mosquitoes than any other effort combined. One caveat to this finding, however, is the potential for misclassification bias; HLC collections were speciated genetically, while other trapping data is generally based on high-throughput morphologic identification, which may fail to identify a species that is not expected in high numbers and is morphologically similar to a common species.

### Interventions and future research

The surprising abundance of *Cx. salinarius* in the HLC collections described here in Chicago is a testament to the effectiveness of the HLC collection method [63]. This study provides compelling evidence in support of alternative trapping methods as a useful tool to provide updates on the overall abundance and diversity of potential disease vectors that are targeting human hosts. As a standard public health measure, these types of environmental health “checkups” may be needed more frequently due to the rapidly changing forces of the present day, such as new introductions of vectors and pathogens.

Public health and mosquito control agencies should consider adding a supplemental plan to their current monitoring and mitigation strategies, occasionally conducting surveillance of and/or targeting natural breeding areas where *Cx. salinarius* may reside (see Additional file 1: Text S1 & Figure S5 for brief habitat analysis). While HLC collection methods are not practical for routine surveillance, sparing usage implemented at strategic time periods (e.g. for early season WNV “sentinel” use, at or around historical peak human transmission, in known “hot spot” human and/or bird transmission locations, etc.) could provide added utility and breadth to existing strategies, providing important information that may increase the efficiency and knowledge in targeting potential mosquito vectors. With the aid of human scented lures and/or CO_2_, traps like BG-sentinels or CDC LTs may also be a viable alternative method for capturing large quantities of *Cx. salinarius* and other human-host seeking mosquitoes in natural habitats [64–66].

While these incidental findings may have implications for the enzootic and zoonotic transmission potential of WNV, suggestions from this study remain as hypotheses and should be interpreted as a guide for improving targeted surveillance and WNV mitigation efforts. Onward research demands the frequent and repeated detection of WNV RNA in *Cx. salinarius* females captured in their environment to substantiate the hypotheses provided in this manuscript. Additional HLC collections should be repeated and include a deeper evaluation of micro-scale factors that may influence a mosquito’s landing preferences, including preferred feeding locations on the human body (Additional file 1: Figure S6), microclimate (Additional file 1: Figure S7), and the addition of sampling residential areas.

## Conclusions

In summary, this research provides an overview of the abundance and generic composition of mosquitoes collected in the Chicago and Decatur, Illinois regions. Traditional GT and LT collections are highly productive, particularly in collecting a broad array of *Culex* species. HLC collection methods clearly showed that *Cx. salinarius* is a potential WNV vector that is in frequent contact with humans in the city of Chicago. The potential of *Cx. salinarius* as a WNV vector in the upper Midwestern USA has been underestimated for two reasons: (i) historically, commonly used traps have only yielded very small fractions of identified *Cx. salinarius*; and (ii) the potential for misidentification of individual species in batch collections is high. However, individuals conducting HLC collection methods are subjected to increased risks of the very mosquito-borne pathogens intended for surveillance, and improvements to personal safety must be implemented before any consistent or systematic use of these methods. This study highlights the added utility of HLC collections as a tool for mosquito surveillance and public health officials to identify current and potential mosquito-borne disease risks.

## Supplementary Information

**Additional file 1: Table** S1. Univariate analysis suggests 4 independent variables may provide unique characteristics in determining the preferred habitat(s) of Cx. salinarius within the NWMAD during the summers of 2018 and 2019. Further analysis is not warranted at this time, given the small number of specimens collected. Values in parenthesis indicate one standard error from the mean. No evidence of multicollinearity was present. **Table** S2. Correlogram testing for multicollinearity among 4 independent variables associated with Cx. salinarius habitat preference within the HLC study regions of NWMAD. Figure S1. Cumulative HLC collections by mosquito genus for each of the 55 hexagons in the study region. **Table S3. **Detailed mosquito collection information by genus, trap type, overall trapping effort and submitting agency. **Table S4. A**Overall number of female mosquitoes collected by trap night for each study location by year, trap type and genus.** B** Trap night information from Southern Cook County was limited to collections by light trap for the years 2005-2008. **Figure S1. **Cumulative HLC collections by mosquito genus for each of the 55 hexagons in the study region. **Figure S2. **Cumulative female mosquito collections by genus from MMAD, NWMAD and Southern Cook County study locations. **Figure S3. **Cumulative female *Culex *spp. collections by light trap or gravid trap from MMAD, NWMAD and Southern Cook County study locations. Only trap type data for *Culex *spp. mosquitoes was available for all 3 collection sources. **Figure S4. **Mosaic plot displaying frequency of genus and species collected by human landing catch by study location (HexID:* n* = 55). For specific coordinates of each mosquito collection location within each HexID, contact the corresponding author. **Figure S5. **Mosaic plot of frequency of mosquito landing location on body of human collector by genus and species. The diagrams on the far right of the figure display the most common landing locations by nuisance (top) and *Culex *species (bottom) mosquitoes. **Figure S6. **Box plot distribution of average weather factors (temperature, humidity, and wind speed) during HLCs by genus and species of collected mosquitoes. Temperature and wind speed were recorded by regionally reported value and by a handheld anemometer/thermometer combination device for specific, local values. **Figure S7. **Suitable larval habitats within the average female *Cx. salinarius *flight range (2.2 km) for each collection from the city of Chicago, MMAD and HLC study regions (55 1-km-wide hexagons within the NWMAD). **Text S1**. Appendix.

## Data Availability

The Chicago-area-HLC data used for analysis in this study are available in the Illinois HLC repository, https://github.com/juel15401/Chicago-area-HLC.git. Due to data privacy and sharing rights, please contact Dr. Gabe Hamer (ghamer@tamu.edu) for Southern Cook County mosquito data, Dr. John-Paul Mutebi (grv0@cdc.gov) for 2005–2006 City of Chicago mosquito data, Dr. Patrick Irwin (pirwin@nwmadil.com) for NWMAD mosquito data, or Jason Probus (JProbus@maconmosquito.org) for MMAD mosquito data.

## Abbreviations

CDC: US Centers for Disease Control and Prevention
HLC: Human landing catch
GT: Gravid trap
IDPH: Illinois Department of Public Health
LT: Light trap
MMAD: Macon Mosquito Abatement District
NWMAD: Northwest Mosquito Abatement District
WNV: West Nile virus
