# Supplementary material for: Human biting mosquitoes and implications for West Nile virus transmission
Source: Parasit Vectors. 2023 Jan 2;16:2. doi: 10.1186/s13071-022-05603-1 (PMC9806905; doi:10.1186/s13071-022-05603-1)
Supplement: Supplementary file 1 — Additional file 1: Table S1. Univariate analysis suggests 4 independent variables may provide unique characteristics in determining the preferred habitat(s) of Cx. salinarius within the NWMAD during the summers of 2018 and 2019. Further analysis is not warranted at this time, given the small number of specimens collected. Values in parenthesis indicate one standard error from the mean. No evidence of multicollinearity was present. Table S2. Correlogram testing for multicollinearity among 4 independent variables associated with Cx. salinarius habitat preference within the HLC study regions of NWMAD. Figure S1. Cumulative HLC collections by mosquito genus for each of the 55 hexagons in the study region. Table S3. Detailed mosquito collection information by genus, trap type, overall trapping effort and submitting agency. Table S4. AOverall number of female mosquitoes collected by trap night for each study location by year, trap type and genus. B Trap night information from Southern Cook County was limited to collections by light trap for the years 2005-2008. Figure S1. Cumulative HLC collections by mosquito genus for each of the 55 hexagons in the study region. Figure S2. Cumulative female mosquito collections by genus from MMAD, NWMAD and Southern Cook County study locations. Figure S3. Cumulative female Culex spp. collections by light trap or gravid trap from MMAD, NWMAD and Southern Cook County study locations. Only trap type data for Culex spp. mosquitoes was available for all 3 collection sources. Figure S4. Mosaic plot displaying frequency of genus and species collected by human landing catch by study location (HexID: n = 55). For specific coordinates of each mosquito collection location within each HexID, contact the corresponding author. Figure S5. Mosaic plot of frequency of mosquito landing location on body of human collector by genus and species. The diagrams on the far right of the figure display the most common landing locations by nuisance (top) and Culex [file 13071_2022_5603_MOESM1_ESM.docx]

**Additional File 1 contains:**

**Tables S1-S4**

**Figure S1-S7**

**Text S1**

**Table S1.** Univariate analysis suggests four independent variables may provide unique characteristics in determining the preferred habitat(s) of *Culex salinarius* within the NWMAD during the summers of 2018 and 2019. Further analysis is not warranted at this time, given the small number of specimens collected. Values in parenthesis indicate one standard error from the mean. No evidence of multicollinearity was present.

| **Independent Variable** | ***Culex salinarius* status** | | ***P*** |
| --- | --- | --- | --- |
|  | **Present** | **Absent** |  |
| percentage black | 4.17 | 1.79 | 0.0205 |
|  | (0.9) | (0.42) |  |
| percentage housing built between 1970-1989 | 52.36 | 34.72 | 0.0321 |
|  | (7.25) | (3.42) |  |
| median household income | $68221.80 | $80706.20 | 0.169 |
|  | ($8098.90) | ($3817.90) |  |
| percentage housing built before World War II | 3.94 | 9.65 | 0.1867 |
|  | (3.86) | (1.82) |  |

|  | **percentage black** | **percentage housing built between 1970-1989** | **median household income** | **percentage housing built before World War II** |
| --- | --- | --- | --- | --- |
| **percentage black** | 1 | 0.374 | -0.244 | -0.436 |
| **percentage housing built between 1970-1989** | 0.374 | 1 | -0.535 | -0.158 |
| **median household income** | -0.244 | -0.535 | 1 | 0.227 |
| **percentage housing built before World War II** | -0.436 | -0.158 | 0.227 | 1 |

**Table S2.** Correlogram testing for multicollinearity among four independent variables associated with *Culex salinarius* habitat preference within the HLC study regions of NWMAD.

**Table S3.** Detailed mosquito collection information by genus, trap type, overall trapping effort, and submitting agency.

**Table S3. (continued).**

**Table S4.** Overall number of female mosquitoes collected by trap night for each study location by year, trap type, and genus (A). Trap night information from Southern Cook County was limited to collections by light trap for the years 2005-2008 (B).

**A.**

| **Study Location** | **Year** | **# Female Culex spp. Collected per Light Trap Night** | **Standard Error** |  |
| --- | --- | --- | --- | --- |
|  |  |  |  |  |
|  |  |  |  |  |
| Southern Cook County, IL | 2005 | 7.50 | 1.49 |  |
|  | 2006 | 15.64 | 3.53 |  |
|  | 2007 | 27.35 | 7.65 |  |
|  | 2008 | 9.52 | 1.10 |  |

**B.**

**
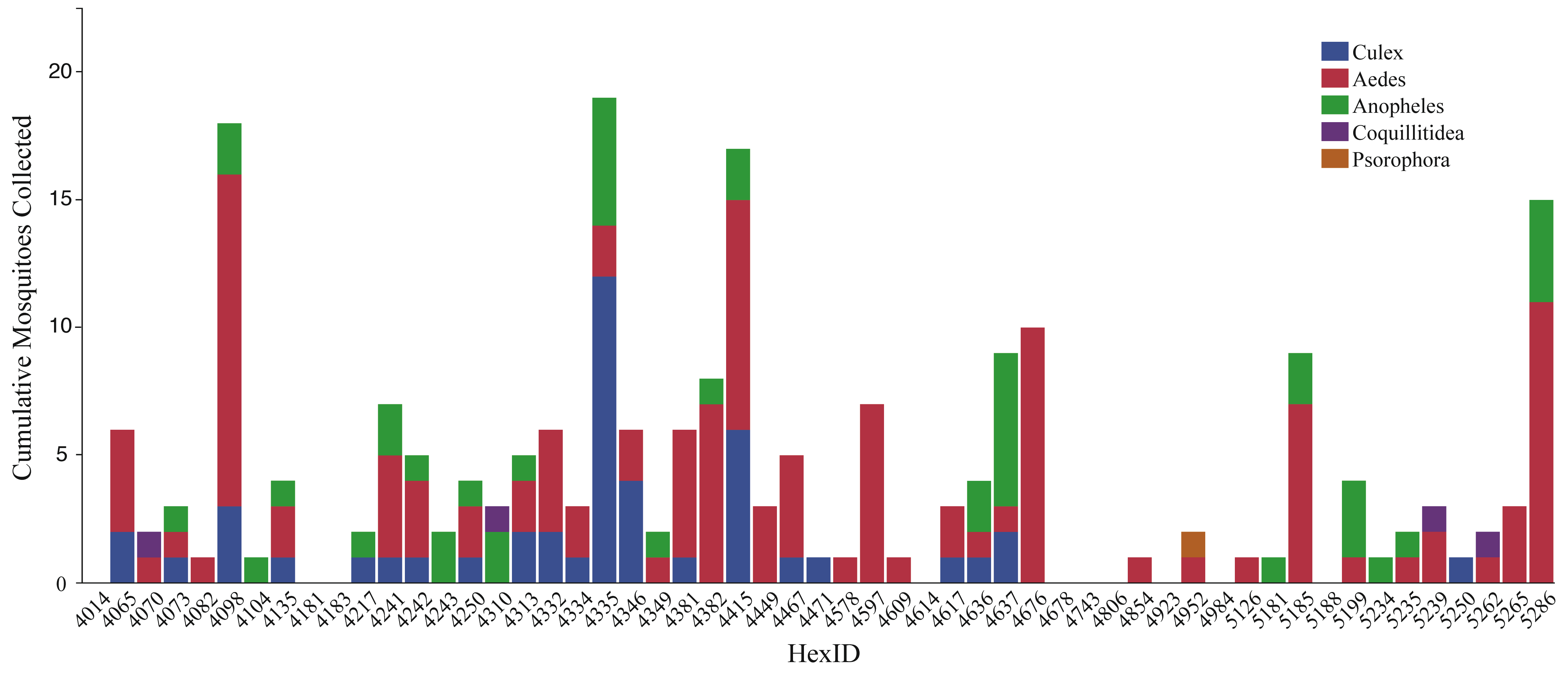
Figure S1.** Cumulative HLC collections by mosquito genus for each of the 55 hexagons in the study region.


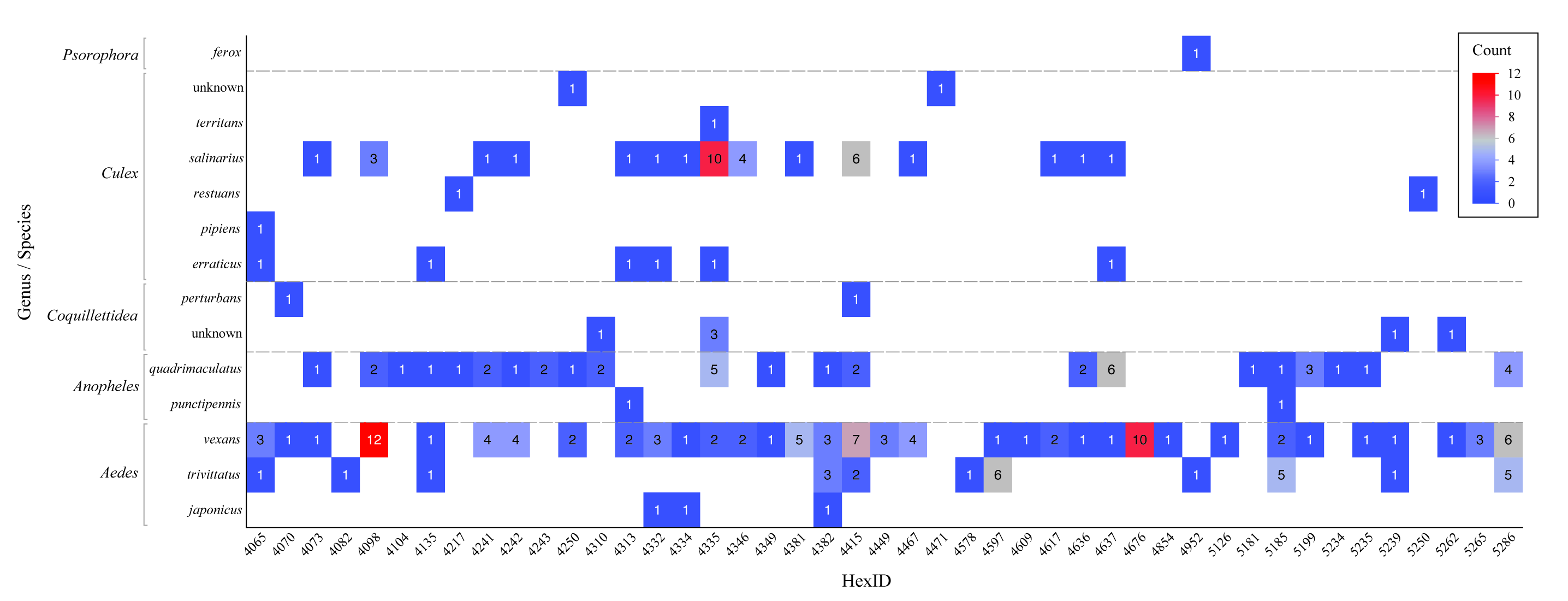
**Figure S2.** Mosaic plot displaying frequency of genus and species collected by human landing catch by study location (HexID, n=55). For specific coordinates of each mosquito collection location within each HexID, contact the corresponding author.

**
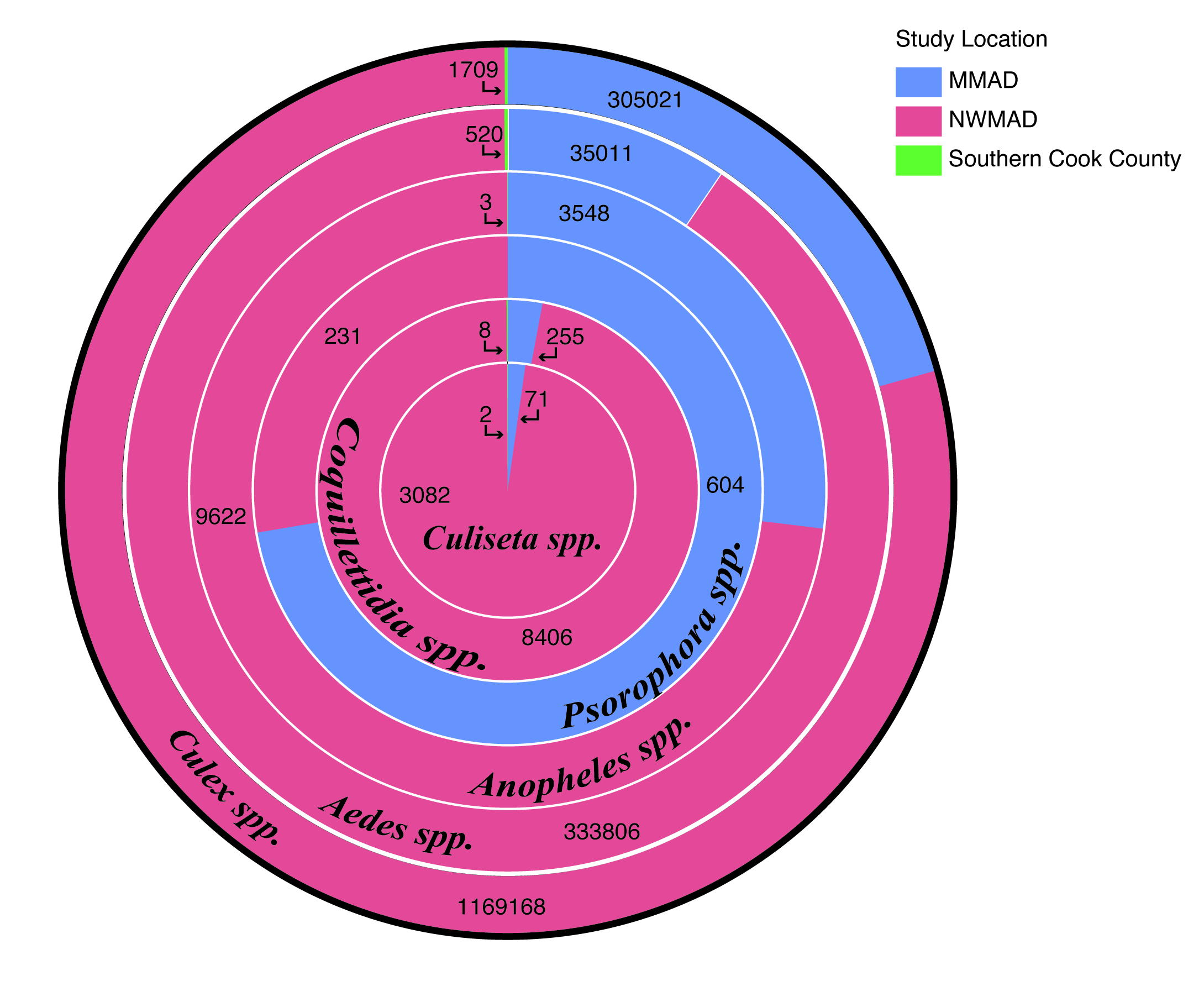
Figure S3.** Cumulative female mosquito collections by genus from MMAD, NWMAD, and Southern Cook County study locations.

**
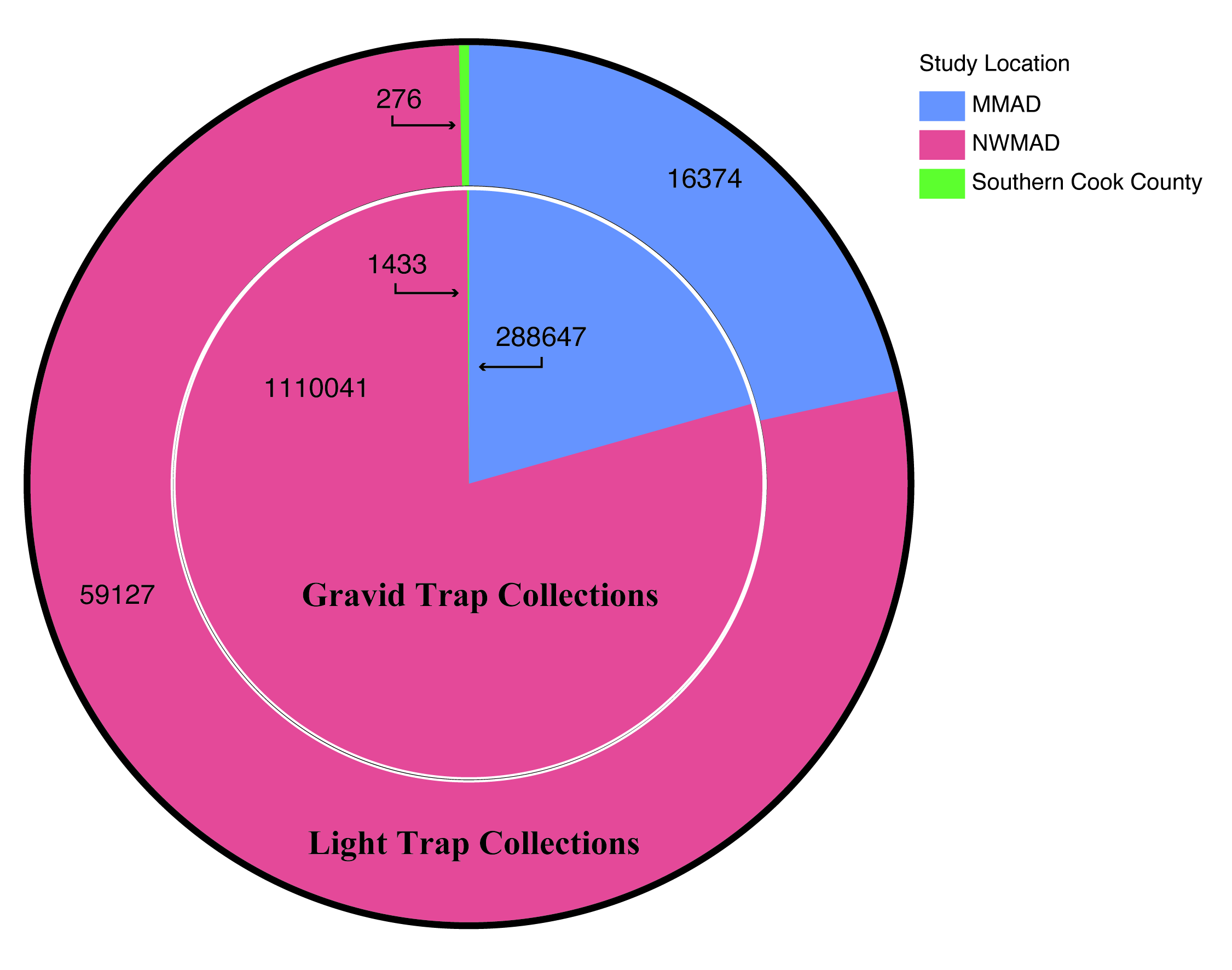
Figure S4.** Cumulative female *Culex* spp. collections by light or gravid trap from MMAD, NWMAD, and Southern Cook County study locations. Only trap type data for *Culex* spp. mosquitoes was available for all three collection sources.

**
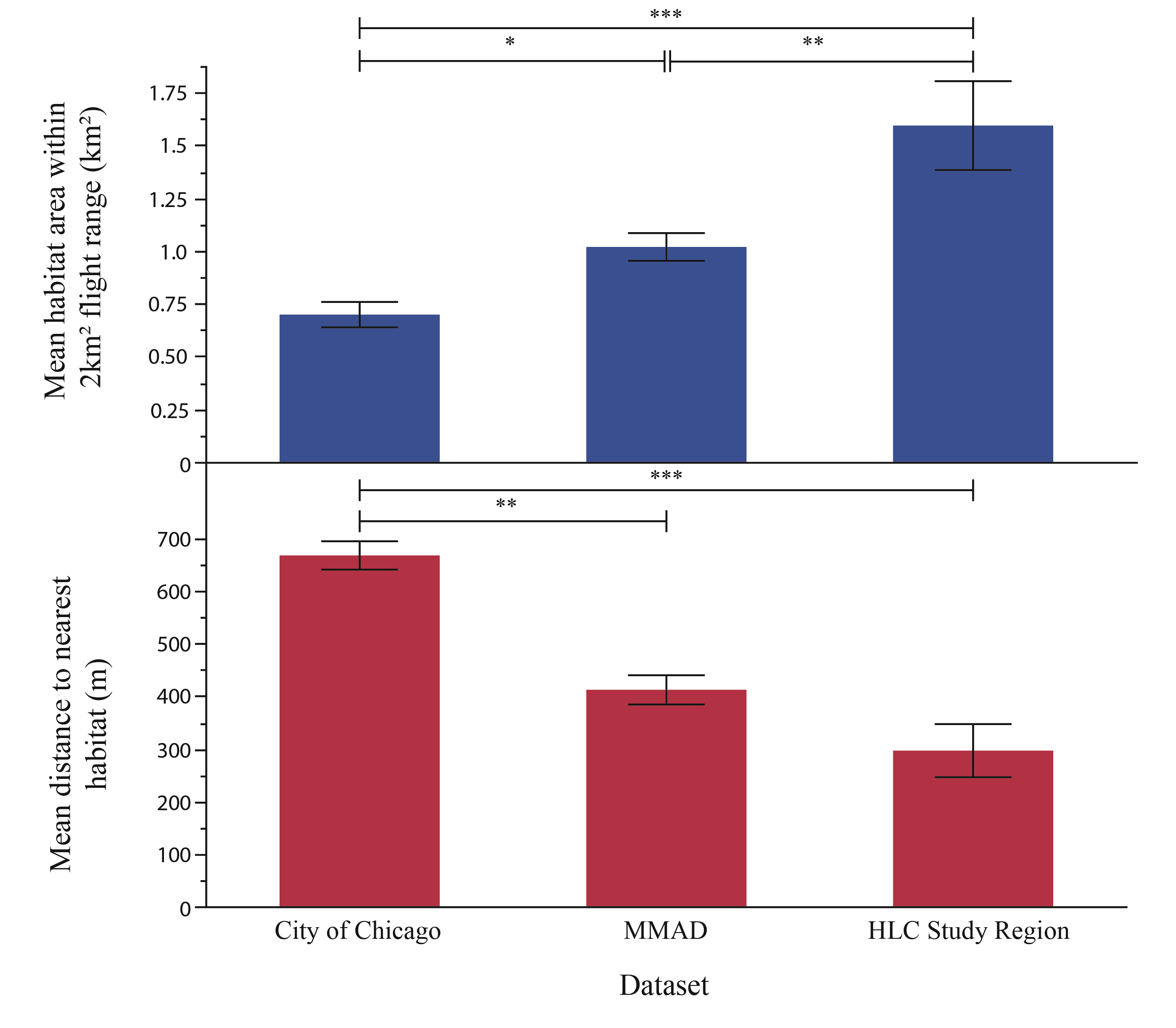
Figure S5.** Suitable larval habitats within the average female *Culex salinarius* flight range (2 km.^2^) for each collection from the City of Chicago, MMAD, and HLC study regions (55 1-km wide hexagons within the NWMAD).


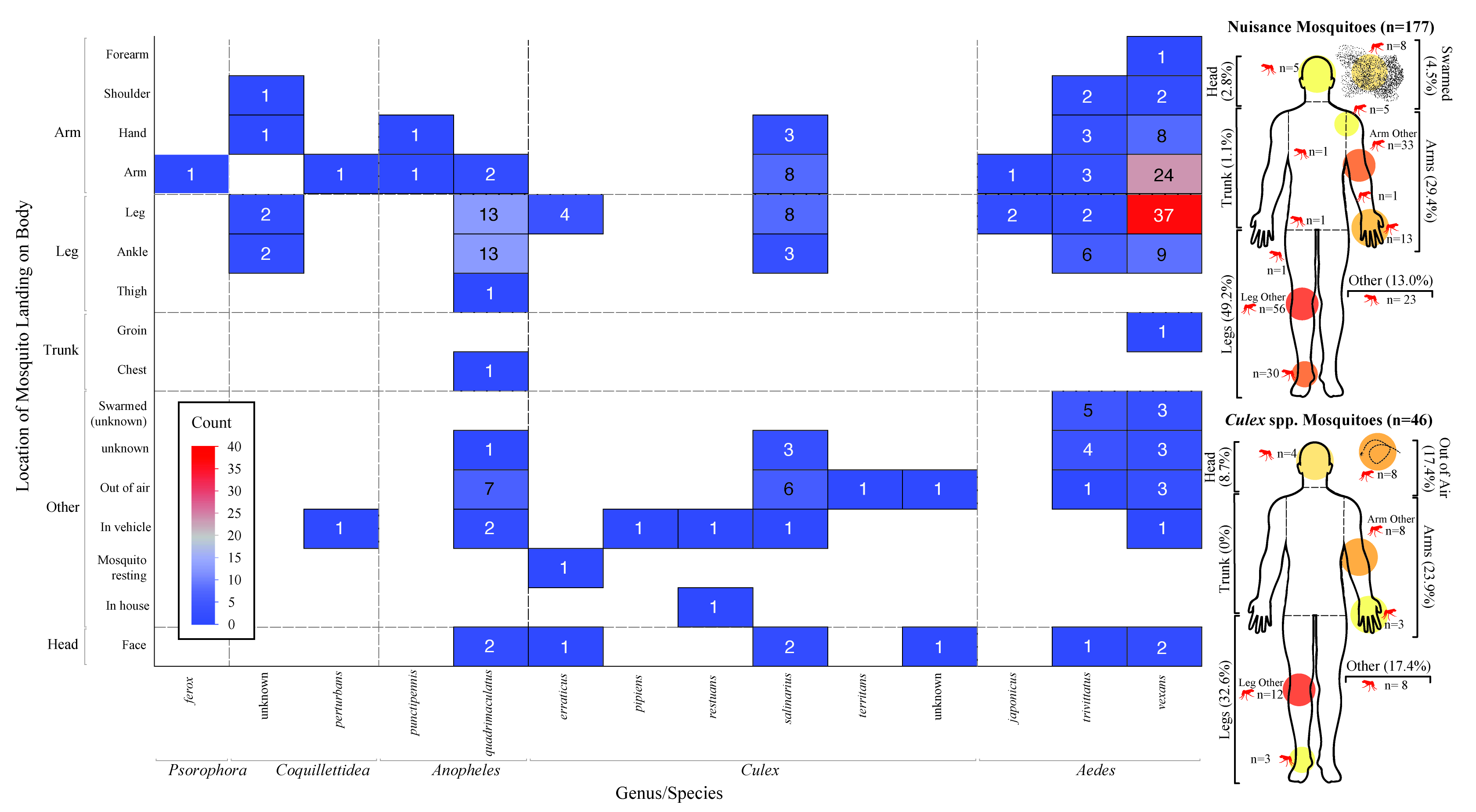
**Figure S6.** Mosaic plot of frequency of mosquito landing location on body of human collector by genus and species. The diagrams on the far right of the figure display the most common landing locations by nuisance (top) and *Culex* species (bottom) mosquitoes.


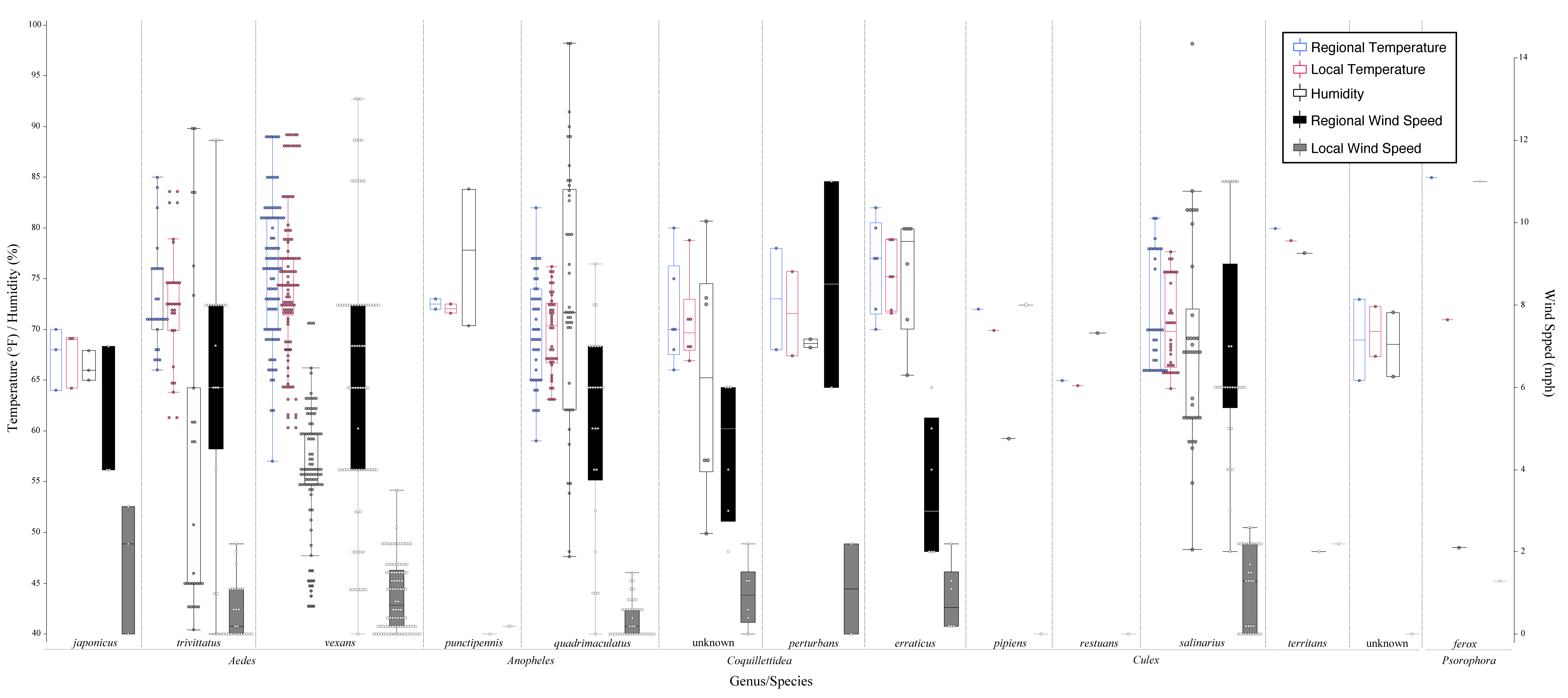
**Figure S7.** Box plot distribution of average weather factors (temperature, humidity, and wind speed) during human landing catches by genus and species of collected mosquitoes. Temperature and wind speed were recorded by regionally reported value and by a handheld anemometer/thermometer combination device for specific, local values.

**Text S1. Appendix.**

*Potential Breeding Habitats of Cx. salinarius in Chicago and Decatur, IL*

In comparison to the traditionally implicated WNV vectors like *Cx. pipiens, Cx. restuans, Cx. tarsalis,* and *Cx.quinquefasciatus, Cx. salinarius* has been understudied. A few previous studies have estimated that the female flight range is likely ~2 km [65]. To quantify potential larval habitats in locations where *Cx salinarius* was collected for this study, high-resolution satellite imagery were overlaid by 2 km buffers around each individually collected mosquito. Each individual natural body of freshwater, including locations with evidence of flooding (e.g. ditches, edges of reservoirs), were traced and converted into polygon shapefiles. Results indicate that locations where *Cx. salinarius* was previously collected have plentiful sources of freshwater (at least 0.75 km^2^), with no mosquito having to travel more than 700 m, a distance well within their average flight range, to reach natural bodies off freshwater (Additional File 1: Figure S7). Additionally, univariate analyses of 40 socio-demographic factors suggests four independent variables may have positive associations with the presence of *Cx. salinarius*: higher % black population (p=0.02), higher % housing built between 1970-1989 (p=0.03), lower median household income (p=0.17), and higher % housing built before WWII (p=0.19) (Additional File 1: Tables S1 & S2).
